# Value of Intraoperative 3D Imaging on the Quality of Reduction of the Distal Tibiofibular Joint When Using a Suture-Button System

**DOI:** 10.1177/10711007221138775

**Published:** 2022-12-20

**Authors:** Fabian T. Spindler, Federico P. Gaube, Wolfgang Böcker, Hans Polzer, Sebastian F. Baumbach

**Affiliations:** 1 Department of Orthopaedics and Trauma Surgery, Musculoskeletal University Center Munich (MUM), University Hospital, LMU, Munich, Germany

**Keywords:** syndesmosis, reduction, intraoperative 3D imaging, suture-button system

## Abstract

**Background:**

The quality of reduction of the distal tibiofibular joint (DTFJ) has a major impact on the outcome. Novel suture-button systems as well as intraoperative 3D imaging can be applied to increase the quality of DTFJ reduction intraoperatively. The individual effect of either remains unknown. The aim of this study was to investigate the value of intraoperative 3D imaging on the quality of reduction of the DTFJ when using a suture-button system.

**Methods:**

Retrospective, radiographic study including adult patients who underwent surgical stabilization of the syndesmosis with a suture-button system for acute, unilateral, unstable syndesmotic injuries with postoperative bilateral CT imaging. The use of an intraoperative 3D scan was the individual surgeon’s choice. Assessed was whether the intraoperative 3D imaging had an influence on the postoperative quality of DTFJ reduction and revision rates. These findings were put in perspective to the correction potential of the suture-button system.

**Results:**

A total of 147 patients were included; 76 of these received an intraoperative 3D imaging. Neither the rate of formal malreduction (17% vs 17%) nor the postoperative revision rate (4% vs 3%) differed significantly between patients with or without intraoperative 3D imaging. Intraoperative 3D imaging revealed a false-negative rate of 14%. The intrinsic correction potential of the suture-button system reduced the number of formally malreduced DTFJs in both groups by 51%.

**Conclusion:**

The additional value of intraoperative 3D imaging to assess the quality of DTFJ reduction in our series did not improve syndesmotic reduction when using a flexible suture-button system.

**Level of Evidence:**

Level III, retrospective comparative cohort study.

## Introduction

Syndesmotic injuries have a reported incidence of 15 per 100 000 and are present in 20% of all operatively treated ankle fractures.^5,14,22^ Syndesmotic malreduction is known to be an independent risk factor for inferior patient-related outcomes and osteoarthritis.^1,9,18^ Previous studies have reported distal tibiofibular joint (DTFJ) malreduction rates of up to 39%.^10,13,19,23,24^ Consequently, there is an ongoing discussion on the best treatment strategy for syndesmotic injuries. Today, this discussion focuses on the type of fixation device, that is, syndesmotic screw vs suture-button system, and the value of intraoperative 3D imaging to assess the quality of reduction of the DTFJ.^3,6,13,17^

The currently available RCTs comparing syndesmotic screws to suture-button systems point toward slightly superior clinical outcomes for dynamic fixation.^2,4,17,19^ One reason is likely the reduced DTFJ malreduction rate.^2,15,17,19,20^ In a previous study, the authors provided data suggesting a natural correction potential of the suture-button system on the DTFJ, which could impact lower malreduction rates.^21^

Intraoperative 3D imaging has been promoted to assess, among others, the quality of DTFJ reduction intraoperatively. Still, its value remains controversial.^3,7,8,11^ Whereas 2 large retrospective trials argue for intraoperative 3D imaging,^3,8^ 2 smaller prospective studies found no additional value of intraoperative 3D imaging on the quality of DTFJ reduction.^7,11^ Consequently, the value of intraoperative 3D imaging to assess the quality of DTFJ remains a matter of debate. When additionally considering the natural correction potential of suture-button systems, the value of intraoperative 3D could be even less.

The aim of this study was to investigate the value of intraoperative 3D imaging on the quality of reduction of the distal tibiofibular joint when a flexible fixation system was used.

## Materials and Methods

A retrospective radiographic study was performed based on a previously published cohort.^21^ The study was approved by the local ethics committee (no. 21-1136).

### Patient selection

**Figure 1.**
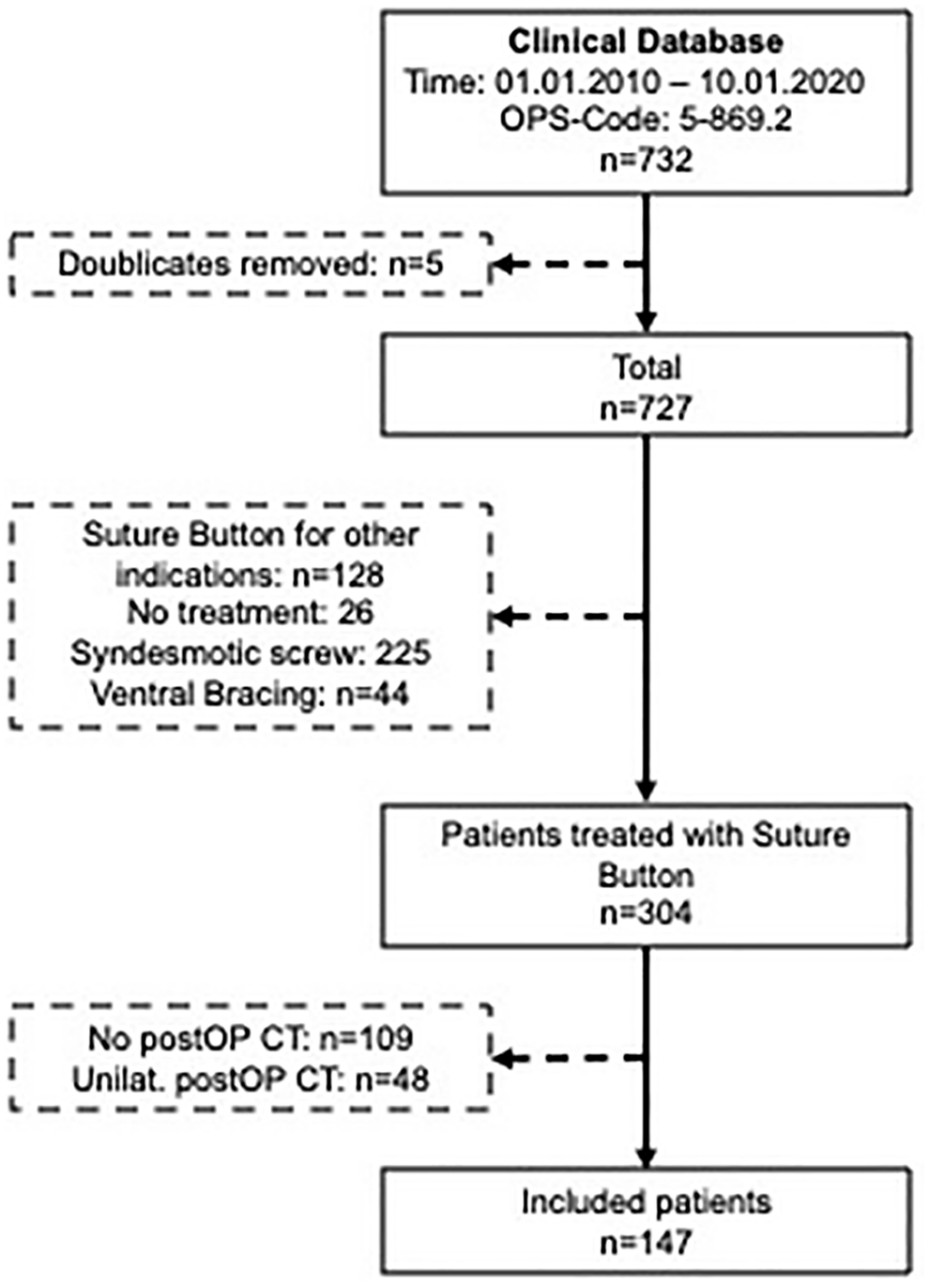
Patient selection. CT, computed tomography; postOP, postoperatively; unilat., unilateral.

The surgical procedure and patient selection (Figure 1) has been described in detail previously.^21^ Overall, 147 adult patients were included from the authors’ ankle fracture database who underwent surgical stabilization of the syndesmosis using a suture-button system for an acute, unilateral ankle injury, with postoperative bilateral CT imaging. Patients with concomitant injuries outside the ankle or a known injury to the contralateral ankle were excluded.

The level 1 trauma facility where the study was carried out has an intraoperative 3D scanner (Ziehm Vision Vario 3D; Ziehm imaging GmbH, Nuernberg, Germany) available. Ankle fractures were treated by 13 different surgeons, but 90% of procedures were performed by 2 senior foot and ankle surgeons (H.P., S.F.B.). The indication for an intraoperative 3D scan is up to the individual surgeon. The scan is typically performed after all bony injuries have been fixed, the DTFJ has been reduced using a reduction forceps applied center-center on the medial and lateral malleolus, and the guide K-wire for the suture-button system has been placed. Then, multiplanar reconstructions of all 3 standard planes (axial, coronal, and sagittal) are generated and evaluated for DTFJ reduction and K-wire positioning.

### Data assessed

Data assessed were general demographics (age, gender, height, weight, body mass index, and American Society of Anesthesiologists class), injury details, intraoperative 3D imaging, intraoperative revision rates, radiation dose and time, quality of DTFJ reduction postoperatively, and postoperative revision rates.

**Figure 2.**
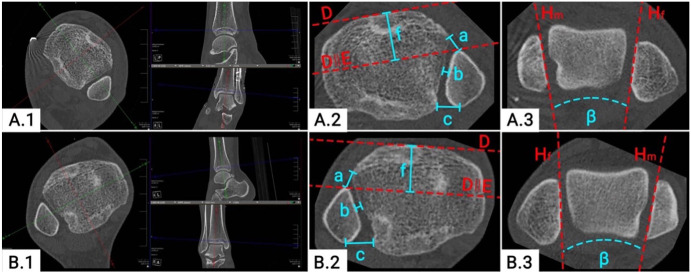
Quality of DTFJ reduction on bilateral CT images. (**A.1**) DTFJ reconstruction of the reduced ankle. (**A.2**) Measurement of the DTFJ reduction of the reduced ankle. (**A.3**) NTDA of the reduced ankle. (**B.1**) DTFJ- reconstruction of the contralateral side. (**B.2**) Measurement of the DTFJ reduction of the contralateral ankle. (**B.3**) NTDA of the contralateral side. a, anterior tibiofibular distance; b, central tibiofibular distance; β, NTDA; c, posterior tibiofibular distance; CT, computed tomographic; D, tangent to the anterior aspect of the tibia; DTFJ, distal tibiofibular joint; E, translation of D to the most anterior part of the fibula; f, sagittal DTFJ-translation of the reduced ankle; H, tangent to the medial malleolus (m) or fibula (f); NTDA, Nault talar dome angle; ||, translation.

All postoperative radiographic measurements were conducted by 2 investigators (F.T.S., S.F.B.) as outlined previously.^21^ The quality of DTFJ reduction was assessed on separately reconstructed axial CT slices of the postoperative bilateral CT images. Assessed were, the anterior, central, and posterior tibiofibular distance, the Nault talar dome angle and the modified sagittal translation (Figure 2).^12,16^ Malreduction was defined as recommended by Kubik et al, that is, an anterior-posterior tibiofibular distance or translation >2 mm, central tibiofibular distance >1.5 mm, or an NTDA >10 degrees.^12^ Patients with at least 1 parameter deviating from these cutoff values were defined as “formally malreduced.” The decision for postoperative revision was based on the individual surgeon’s decision and not based on the formal measurements of the DTFJ reduction.

**Figure 3.**
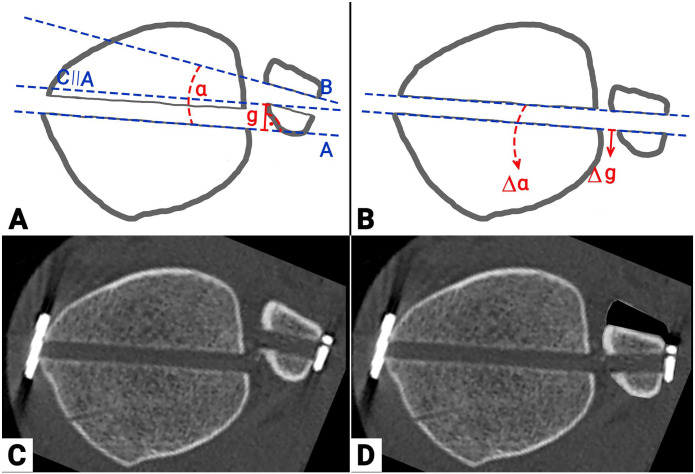
Illustration of the recalculation procedure for the intraoperative trilling tunnel alignment based on the postoperative computed tomographic imaging. A: Schematic drawing of postopertive measurments for axial translation (g) and rotation (a) of the suture-button drilling tunnel. (A) Posterior border of the tibial drill tunnel (B) Anterior border of the fibial drill tunnel (C) Line parallel to A through the most medial aspect of posterior fibular drill tunnel B: Recalculated intra-operative reduction of the distal tibio-fibular joint by aligning the tibial and fibular drill tunnels. C: Postoperative CT plane through the drill tunnel. D: Illustration of the intraoperative reduction based on the alignement of the tibial and fibular drill tunnels.

According to the standardized protocol of the previous trial, the initial, intraoperative reduction was recalculated.^21^ Based on the postoperative CT imaging, the axial translation (Figure 3A, g) and rotation (Figure 3A, a) of the suture-button drilling tunnel were measured. It can be assumed that at the point of drilling for the suture-button system, the drilling tunnels in the tibia and fibula must have been aligned. Consequently, the axial translation (Figure 3B, g) / rotation (Figure 3B, a) were subtracted from the above-outlined postoperative CT measurements, that is, the modified sagittal translation / the Nault talar dome angle. These adjusted values were again compared to the healthy, uninjured side and the quality of DTFJ reduction was recalculated, as outlined above.

### Statistics

Data were analyzed using IBM Statistical Package for the Social Sciences, version 28 (SPSS). Descriptive statistics, independent samples *t* tests, and χ^2^ tests were conducted. If not stated differently, statistical values are presented as mean ± SD. Statistical significance was set as *P* values lower than .05.

## Results

The patients’ mean age was 39.3±14.8 years, their body mass index was 26.3±4.8, and 34% were female; 63.3% suffered an ankle fracture and the remaining patients an isolated syndesmotic injury. No side differences were observed (50% left). An intraoperative 3D scan was intended in 82 patients. In 6 patients, the scan could not be conducted as planned, because of technical errors prior to the scan (n=4) or technical errors after the scan (n=2). The remaining 76 patients (52%) formed the intraoperative 3D imaging group (Table 1). Those 6 patients with a failed scan were added to the group of patients that had not received an intraoperative 3D scan.

**Table 1.** Demographics per the Different Groups.

	Intraoperative 3D Imaging (n = 76; 52%)	No Intraoperative 3D Imaging (n = 71; 48%)	*P* Value ^a^
Age, y, mean ± SD	36.5±13.8	42.3±15.4	**.019**
BMI, mean ± SD	26.7±5.7	25.9±4.0	.394
Injury			**<.001**
Fracture	34	59	
Syndesmosis	42	12	
Percentage of isolated syndesmotic injuries	55	17	**<.001**
Fractures according to the AO Classification	A2: 0; B1: 6; B2: 6; B3: 4; C1: 2; C2: 10; C3: 3; Not classified: 3	A2: 1; B1: 7; B2: 5; B3: 30; C1: 3; C2: 10; C3: 2; Not classified: 1	**n.a.**
Time of radiation, min	1.28±0.7	0.53±0.6^b^	**<.001**
Radiation dose, cGy/cm^2^	73.4±33.4	26.7±16.3^b^	**<.001**

Abbreviations: AO, arbeitsgemeinschaft osteosynthese; BMI, body mass index; n.a., not assessable.

a Bold values indicate significant differences.

b Values excluding 2 patients who had received a failed 3D scan.

Patients with intraoperative 3D imaging were on average significantly younger (36.5 vs 42.3 years; *P* = .019) and significantly more frequently suffered an isolated syndesmotic injury (55% vs 17%; *P* < .001), but the fracture cases tended to be less severe (AO type B3 and C3: 21% vs 54%). Radiation time and dose were significantly higher in the 3D imaging group.

**Figure 4.**
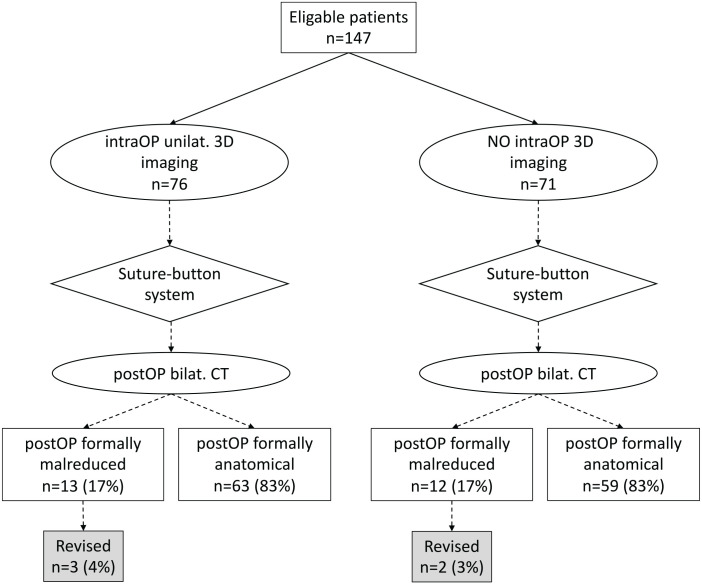
Flowchart comparing formal malreduction rates and revision cases per the use of an intraoperative 3D scan. Bilat., bilateral; CT, computed tomography; IntraOP, Intraoperative; PostOP, Postoperative; Unilat., unilateral.

A groupwise comparison flowchart illustrating the number of formally malreduced DTFJs and the postoperatively revised patients is illustrated in Figure 4. Overall, the application of an intraoperative 3D imaging did not result in a reduction of formally malreduced DTFJ (17% vs 17%). Furthermore, it did not reduce the rate of revision surgery due to malreduction when compared to the group of patients without 3D imaging (4% vs 3%).

Eleven of 76 patients with an intraoperative 3D scan were intraoperatively revised. Of those 11 patients, 4 were identified postoperatively as still malreduced, 1 of which underwent secondary revision surgery. Sixty-five patients had an intraoperative scan that was considered unsuspicious. Out of these, 9 patients were identified postoperatively as malreduced, 2 of which were revised. Comparing patients with an unsuspicious intraoperative 3D scan (n = 65) to those who had no scan intraoperatively (n = 71), revealed no difference in malreduction (14% vs 17%) or postoperative revision rate (3% vs 3%).

As reported previously, the suture-button system itself has the potential to correct toward a more anatomically reduced DTFJ.^21^ When recalculating the intraoperative reduction based on the suture-button drilling tunnel, the number of malreduced DTFJ dropped by 51% after installing the suture-button system, regardless of whether an intraoperative 3D scan was performed or not (Table 2).^21^ Noteworthy, this decrease was considerably lower in those patients who underwent intraoperative revision based on the 3D scan, compared with those that were rated intraoperatively as unsuspicious (33% vs 57%).

**Table 2.** Comparison of the Intraoperative Recalculated to the Postoperative DTFJ Reduction.

	Intraoperative 3D Imaging		No Intraoperative 3D Imaging	
	Malreduced DTFJ	Anatomical DTFJ	Malreduced DTFJ	Anatomical DTFJ
Intraoperatively recalculated DTFJ	27 (36%)	49	24 (34%)	47
Postoperative DTFJ	13 (17%)	63	12 (17%)	59

Abbreviation: DTFJ, distal tibiofibular joint.

## Discussion

The primary aim of this study was to assess the potential additional value of an intraoperative 3D scan on DTFJ malreduction rates in patients treated with a suture-button system. The value of intraoperative 3D imaging for DTFJ malreduction remains a matter of debate.^3,7,8,11^ Franke et al^8^ and Beck et al^3^ each published a large retrospective case series investigating the value of intraoperative 3D imaging in ankle fracture cases with syndesmotic screw stabilization. They reported considerable variation with intraoperative DTFJ malreduction rates of 7% and 26%.^3,8^ However, only 1 of these studies conducted postoperative bilateral CT imaging^3^ and reported that no patient revealed a “defective position of the fibula in the tibial incisura worth revision.” Whether any of these cases was formally malreduced remains unclear. In conclusion, both studies favored intraoperative 3D imaging in case of DTFJ stabilization.

Davidovitch et al^7^ and Kortekangas et al^11^ published 2 trials on smaller patient cohorts. Both studies conducted postoperative bilateral CT imaging. Using intraoperative 3D imaging (n=16) and standard radiographs (n=20) and syndesmotic screws, Davidovitch et al^7^ reported high rates of syndesmotic malreductions for both groups. Kortekangas et al^11^ randomized for suture-button system (n=21) and syndesmotic screw (n=22), with both groups having intraoperative 3D imaging. Postoperatively, 1 patient in each group was malreduced. Intraoperative 3D imaging suggested malreduction in 7 cases in the suture-button group, all of which were anatomically reduced per the postoperative bilateral CT imaging. These 2 studies questioned the additional value of intraoperative 3D imaging.

One of the main objectives of an intraoperative 3D scan is to avoid revision surgery due to a malreduced DTFJ. Overall, 17% of the patients in the present study were judged by our strict criteria to be malreduced, with 3% requiring revision surgery, independent of whether a 3D scan was performed or not. Even if the DTFJ reduction was rated as sufficient by intraoperative 3D imaging, the rates of formal malreduction (14% vs 17%) and postoperative revision surgery (3% vs 3%) did not differ between those patients who had intraoperative 3D imaging and those who did not. In published studies of postoperative bilateral CT imaging to assess DTFJ malreduction, the formal malreduction rates range from 5% to 28%.^7,11^ Two studies found no patient “worth revision” and 1 did not state the actual revision rate.^3,7,11^ Interestingly, 2 of these 3 studies found no additional value of intraoperative 3D imaging.^3,7,11^ Consequently, it remains questionable if intraoperative 3D imaging does reduce the actual number of revision surgeries.

Several factors might affect the validity of intraoperative 3D imaging. First, the current study found a false-negative rate of 14% for the intraoperative 3D scan, with an unknown false-positive rate. Consequently, the conclusion by Beck et al^3^ that intraoperative 3D imaging can replace postoperative bilateral CT imaging remains questionable. Second, 3 of the 4 studies mentioned so far facilitated unilateral intraoperative 3D imaging.^3,7,8^ Only 1 study conducted a bilateral intraoperative 3D scan.^11^ However, the quality of DTFJ reduction can only sufficiently be judged in side-to-side comparison to the contralateral, healthy side.^12^ Noteworthy, even the study conducting bilateral intraoperative 3D imaging voted against its value if a suture-button system is used.^11^ Obviously, the syndesmotic stabilization device also has an effect on the postoperative DTFJ reduction.^21^ Although a syndesmotic screw rigidly fixes the fibula to the tibia in the initially reduced position, a suture-button system provides a dynamic stabilization of the DTFJ, which has been shown to often have a natural correction potential, positively affecting the postoperative quality of DTFJ reduction.^21^ Finally, the actual quality of the intraoperative 3D imaging is highly dependent on the amount of osteosynthetic material implanted as well as the scanner and software used.^8^ Consequently, the actual value of an intraoperative 3D scan remains unknown and must be interpreted together with the syndesmotic stabilization device used.

In the herein presented study, intraoperative 3D imaging did neither reduce the number of formally malreduced DTFJs nor the number of patients necessitating revision surgery. However, the use of a suture-button system reduced the formal malreduction rate from 36% (with 3D imaging) and 34% (without 3D imaging) intraoperatively to, respectively, 17% and 17% postoperatively. In a best-case scenario, 7 of 76 patients in the 3D imaging group might have been saved from postoperative malreduction. Conversely, intraoperative 3D imaging was false negative in 9 cases. Based on the above-mentioned arguments, it appears questionable whether all of these 7 patients were actually saved from malreduction because of the intraoperative 3D scan. Based on the numbers available, the odds of an anatomically reduced DTFJ appear to be considerably more influenced by the choice of the fixation device than by the use of an intraoperative 3D scan.

Assessing DTFJ reduction is only one of the aspects of intraoperative 3D imaging. It can also be used to assess the quality of bony reduction in complex fracture cases or to identify intra-articular loose bodies.^3^ However, the additional value per the different indications for intraoperative 3D imaging must be weighed against the increased radiation exposure. In the currently performed trial, the radiation exposure of patients who received intraoperative 3D imaging was significantly higher compared with standard intraoperative fluoroscopy (73.4 vs 26.7 cGy/cm^2^; *P* < .001).

Several limitations must be discussed. First, a clear limitation relates to the retrospective study design. We have the possibility of selection bias for the application of an intraoperative 3D scan per the type of injury. Although patients with intraoperative 3D imaging were on average younger and more often suffered an isolated injury to the syndesmotic complex, the fracture cases were less frequent (45% vs 83%) and less severe (7 Arbeitsgemeinschaft Osteosynthese [AO] type B3 or C3 fractures vs 32 AO type B3 or C3 fractures) compared with those patients who had no intraoperative 3D imaging. As a result, selection bias may be based on the patients’ age rather than the complexity of the fractures. We have reanalyzed the overall frequency of intraoperative 3D imaging over the time course and the influence of the treating surgeon for an intraoperative 3D imaging. Whereas an intraoperative 3D scan was performed in 80% of cases in 2015, this number decreased to about 20% in 2020. This change could be interpreted as a learning curve for the suture-button system, which was implemented in 2015. Moreover, 90% of the surgeries were conducted by one of 2 senior foot and ankle surgeons. There was no equity in the deployment of 3D imaging. One surgeon used 3D intraoperative imaging for 80% of his cases, the other did so in only 29% of his cases. The distribution of fracture and isolated syndesmotic injury cases was rather homogeneous between these 2 surgeons. Consequently, the type of injury was likely not a confounder. However, it is possible that patients’ age, a potential learning curve, and the preferences of the treating surgeon could have introduced some bias in the use of an intraoperative 3D scan.

Finally, the time of intraoperative 3D imaging might have an influence on its diagnostic value. In the current study, the 3D scan was performed prior to the actual implantation of the suture-button system. All previously studies conducted the scan just prior to wound closure.^3,7,8,11^ The time of scanning most likely has no influence when a static fixation device is used, but might mislead in the setting of a suture-button system.^11^ The downside of conducting the scan just prior to wound closure is that a possible revision of the DTFJ and re-placement of the syndesmotic stabilization device is more complicated because of the preexisting drilling hole. The authors therefore prefer to perform the 3D scan with just the guidewire in place.

In conclusion, the additional value of intraoperative 3D imaging to assess the quality of DTFJ reduction appears questionable if a suture-button system is used. Because of a considerable rate of false-negative findings, it also does not preclude incremental-value postoperative bilateral CT imaging to understand the anatomic outcome of surgical stabilization. In terms of a risk-benefit consideration, we now conduct intraoperative 3D imaging when a suture-button system is used only in isolated syndesmotic injury with a ligamentous rupture of all 3 syndesmotic ligaments (anterior-inferior tibiofibular ligament, interosseous ligament, posterior-inferior tibiofibular ligament), pilon fractures to assess the quality of bony reduction, or revision cases.

## Supplemental Material

sj-pdf-1-fai-10.1177_10711007221138775 – Supplemental material for Value of Intraoperative 3D Imaging on the Quality of Reduction of the Distal Tibiofibular Joint When Using a Suture-Button System Click here for additional data file. Supplemental material, sj-pdf-1-fai-10.1177_10711007221138775 for Value of Intraoperative 3D Imaging on the Quality of Reduction of the Distal Tibiofibular Joint When Using a Suture-Button System by Fabian T. Spindler, Federico P. Gaube, Wolfgang Böcker, Hans Polzer and Sebastian F. Baumbach in Foot & Ankle International
